# Transformer-based ECG classification for early detection of cardiac arrhythmias

**DOI:** 10.3389/fmed.2025.1600855

**Published:** 2025-08-22

**Authors:** Sunnia Ikram, Amna Ikram, Harvinder Singh, Malik Daler Ali Awan, Sajid Naveed, Isabel De la Torre Díez, Henry Fabian Gongora, Thania Candelaria Chio Montero

**Affiliations:** ^1^Department of Software Engineering, The Islamia University of Bahawalpur, Bahawalpur, Pakistan; ^2^Faculty of Computing, The Govt Sadiq College Women University, Bahawalpur, Pakistan; ^3^Department of Mechanical Engineering, Chandigarh Group of Colleges, Landran, Mohali, Punjab, India; ^4^Department of Signal Theory and Communications and Telematics Engineering, University of Valladolid, Valladolid, Spain; ^5^Universidad Internacional Iberoamericana, Campeche, Mexico; ^6^Universidad Internacional Iberoamericana, Arecibo, PR, United States

**Keywords:** cardiac monitoring, ECG classification, electrocardiogram analysis, PCA, t-SNE, Transformer-based model, VPC, feature engineering

## Abstract

Electrocardiogram (ECG) classification plays a critical role in early detection and trocardiogram (ECG) classification plays a critical role in early detection and monitoring cardiovascular diseases. This study presents a Transformer-based deep learning framework for automated ECG classification, integrating advanced preprocessing, feature selection, and dimensionality reduction techniques to improve model performance. The pipeline begins with signal preprocessing, where raw ECG data are denoised, normalized, and relabeled for compatibility with attention-based architectures. Principal component analysis (PCA), correlation analysis, and feature engineering is applied to retain the most informative features. To assess the discriminative quality of the selected features, t-distributed stochastic neighbor embedding (t-SNE) is used for visualization, revealing clear class separability in the transformed feature space. The refined dataset is then input to a Transformer- based model trained with optimized loss functions, regularization strategies, and hyperparameter tuning. The proposed model demonstrates strong performance on the MIT-BIH benchmark dataset, showing results consistent with or exceeding prior studies. However, due to differences in datasets and evaluation protocols, these comparisons are indicative rather than conclusive. The model effectively classifies ECG signals into categories such as Normal, atrial premature contraction (APC), ventricular premature contraction (VPC), and Fusion beats. These results underscore the effectiveness of Transformer-based models in biomedical signal processing and suggest potential for scalable, automated ECG diagnostics. However, deployment in real-time or resource-constrained settings will require further optimization and validation.

## 1 Introduction

Electrocardiography is a primary and most used technique in cardiology that records electrical signals of the heart and analyzes the state of the heart. The increasing number of patients with CVDs, arrhythmia, myocardial infarction and heart failure proves that accurate and reliable diagnostic tools are needed (1). The initial stages of automated ECG classification were supported by convolutional models, which provided high accuracy and efficiency, although they typically relied on fixed-size kernels and local feature extraction (2). As such, there is a growing need for automated ECG classification systems that can efficiently assist clinical decision-making and improve the quality of diagnostic results.

In recent years, the global incidents of CVDs has increased, making them one of the leading causes of death worldwide (3). ECG as a non-invasive technique is widely used for diagnosing cardiac arrhythmia and abnormalities. Prodromal signs of CVDs often manifest as irregular electrical patterns, detectable via ECG signals. For instance, cardiac arrhythmias can be fatal if not monitored properly, as they may indicate conditions leading to sudden cardiac arrest. Acharya et al. (4) employed CNN-based architectures to classify ECG signals and achieved 95% accuracy. Similarly, Liu et al. proposed the RNN-based approaches, demonstrating the ability of sequence-based models to capture temporal dependencies, achieving 95% accuracy in arrhythmia classification (5–7). These results indicate that deep learning models are well-suited for ECG classification.

The ability to differentiate between normal and arrhythmic ECG signals is critical for improving CVD diagnosis and identification (8). However, due to small amplitude variations and short- duration signals, ECG classification remains challenging. Additionally, inherent differences in ECG patterns across different CVDs, and difficulty in distinguishing similar features between patients make classification even more complex. As a result, deep learning-based automated diagnostic tools are crucial in complementing traditional ECG analysis to improve accuracy and efficiency in CVD detection. Chang and Limon (9) demonstrated that transformers could effectively classify ECG signal by focusing on the most relevant signal characteristics using the attention mechanism. Transformers can capture long-range dependencies in ECG measurements well-suited for complex classification tasks.

Building upon these advancements, this study proposes a novel Transformer-based model for multi-class ECG classification, specifically targeting five distinct classes: Normal, APC, VPC, Fusion beat and others. To enhance classification performance, a Transformer-based model is trained on refined ECG features rather than raw ECG signals, enabling better features extraction and reducing noise interference. The model is trained and tested on a publicly available ECG dataset, demonstrating its effectiveness in classifying various cardiac pathologies. To further evaluate the model’s performance, various evaluation metrics are used, ensuring its reliability in real-world applications. Motivations behind this work are:

- Variability of ECG waveforms across individuals due to age, physical condition and emotional state, making it challenging to distinguish between normal and abnormal rhythms.
- Arrhythmic events often have low amplitude and short duration, making them difficult to identify amidst noise.
- Distinguishing between automatically and mechanically mediated arrhythmias remains ambiguous due to overlapping signal characteristic.
- Bio-noise, such as muscle contractions or improper electrode placement, increases signal distortion, affecting classification accuracy.
- Traditional convolutional methods used for noise reduction may also remove critical ECG features, impacting arrhythmia detection.

The analysis of electrocardiogram (ECG) data now generates better results for recognizing heart rhythm irregularities together with better classification of cardiac conditions. Modern approaches solve many problems of traditional techniques through direct ECG signal analysis which removes the requirement for human involvement (10). Recent systems, such as Transformer-based architectures, build upon CNN strengths by enabling long-range dependency modeling and adaptive attention, which enhances recognition of subtle and infrequent ECG patterns (11–13).

These approaches demonstrate strong capabilities in detecting relationships throughout long duration within ECG recordings. Their ability to detect irregular heartbeats that appear infrequently makes these methods highly effective (14, 15). The ensured reliable operation across different patient groups and improved diagnostic accuracy comes from this approach’s capabilities. Real-world ECG measurements do not affect these systems because they demonstrate enhanced resistance to both interference and measurement distortions.

The ability to understand model prediction processes through these techniques increases the potential for medical practitioners to adopt the model. Transformers are particularly well-suited for capturing long-range temporal dependencies across ECG sequences, complementing the local feature extraction of CNNs.

This paper is organized:

- Section 1 presents the Literature Review.
- Section 2 describes Methodology, including data preprocessing, feature selection and model training.
- Section 3 presents the Results and Analysis, where classification outcomes are evaluated.
- Section 4 discusses Findings, Limitations and Future Research Directions.

## 2 Literature review

The identification and classification of cardiovascular disease (CVDs), particularly arrhythmia, remain critical areas of research due to the pivotal role of electrocardiography (ECG) in diagnosing heart disorder. Over the past few decades, various methodologies have been employed for ECG-based arrhythmia detection, ranging from classical machine learning techniques to advanced deep learning approaches, with the primary objective of enhancing accuracy, efficiency and robustness. Martis et al. (16) proposed an SVM-based classification method that relied on handcrafted features such as wavelet coefficients and heart rate variability, as discussed in Table 1. Similarly, Marinho et al. (17, 18) explored feature engineering techniques to improve arrhythmia classification. However, these models exhibit poor generalization on large databases due to their dependence on manual feature extraction, making them highly sensitive to noise and variation in patients.

**Table 1:** State-of-the-art methods for ECG classification.

References	Techniques	Goals	Findings
Lee and Shin (30)	Hierarchical Transformer	Lead-aware ECG modeling	High-performance arrhythmia detection
Hannun et al. (31)	deep neural network (DNN)	improve the accuracy and scalability	reduce the rate of misdiagnosed
Rajpurkar et al. (32)	CNN	exceeds the performance	Exceed cardiologist performance
Arabi et al. (19)	MSW-Transformer	Multi-scale attention ECG classifier	Macro-F1: 77.85%
Ait Bourkha et al. (33)	DCETEN (1D-CNN + Transformer)	Efficient ECG classification	Accuracy: 99.84%
Kailan et al. (34)	PSO-based feature selection + SVM, KNN, RF, DT	Improve ECG classification accuracy & reduce dimensionality for IoT deployment	Accuracy: 98% (PSO-SVM) vs. 84% (non-PSO); Features reduced: 4000 → 888
Mavaddati (35)	ResNet-34 + Time–Frequency Scalogram + Transfer Learning	Classify 3 types of cardiovascular diseases (CVDs); compare with CNN, RNN, SNMF	ResNet-34 outperformed CNN, RNN, and SNMF in accuracy, sensitivity, and robustness for clinical use

To address the limitations of early rule-based and statistical ECG analysis methods, Hannun et al. (15) explored recurrent neural networks (RNNs) and LSTM architectures to preserve temporal information over longer durations. While LSTMs improved arrhythmia classification, they often struggled with vanishing gradient problems and incurred high computational costs—posing a challenge for real-time or resource-constrained deployment.

Transformer models, originally introduced for natural language processing, have recently gained traction in biomedical signal processing due to their ability to model long-range dependencies efficiently. In one of the earliest applications of Transformers to ECG signals, a 2021 study (19) demonstrated their effectiveness in arrhythmia classification. Li et al. (14) further extended this by integrating a Transformer with a 2D-UNet architecture to capture both spatial and temporal ECG features, improving classification accuracy and interpretability.

Despite their promise, Transformers also come with challenges. Training large-scale Transformer models demands significant computational resources and careful hyperparameter optimization. Additionally, their integration into clinical workflows requires further work on improving interpretability and operational efficiency. The contribution of our work is as follows:

- The proposed Transformer-based model was evaluated on five ECG arrhythmia classes: Normal, APC, VPC, Fusion Beat, and Others demonstrating its effectiveness in multi-class ECG classification tasks.
- The model exploits the attention mechanism to learn long-range temporal dependencies, offering improved performance over conventional CNN and RNN approaches.
- It addresses key challenges in ECG analysis, such as noise and signal variability, by focusing on clinically informative signal segments.
- While deployment in clinical settings remains a future goal, the model shows promise for scalable and automated ECG analysis, suitable for integration into health-monitoring systems.

Despite notable advancements in CNNs, LSTMs, and Transformer-based techniques, several key challenges persist. These include limited generalizability across datasets, vulnerability to signal artifacts, and the computational intensity required for model training and inference. Overcoming these obstacles is essential for creating robust, interpretable, and deployable ECG classification systems suitable for real-world clinical use.

## 3 Materials and methods

The proposed ECG classification framework is designed to detect and categorize cardiac arrhythmia using a Transformer-based deep learning model trained on preprocessed ECG signals. The system integrates data acquisition from a wearable device, such as a smartwatch, with a mobile application that transmits ECG data to a cloud server via Wi-Fi for further processing. Upon receipt, the raw ECG signals undergo a structured preprocessing pipeline that includes denoising to eliminate motion artifacts and baseline drift, normalization to standardize signal amplitude, and segmentation to extract uniform time windows for analysis.

Following preprocessing, feature extraction and selection are conducted using techniques such as principal component analysis (PCA) and correlation-based filtering to identify the most discriminative signal characteristics. These selected features serve as input to the Transformer-based architecture, which is trained in the cloud environment using supervised learning. The training phase incorporates hyperparameter tuning, loss function optimization, and regularization strategies to improve generalization and mitigate overfitting.

**Figure 1:**
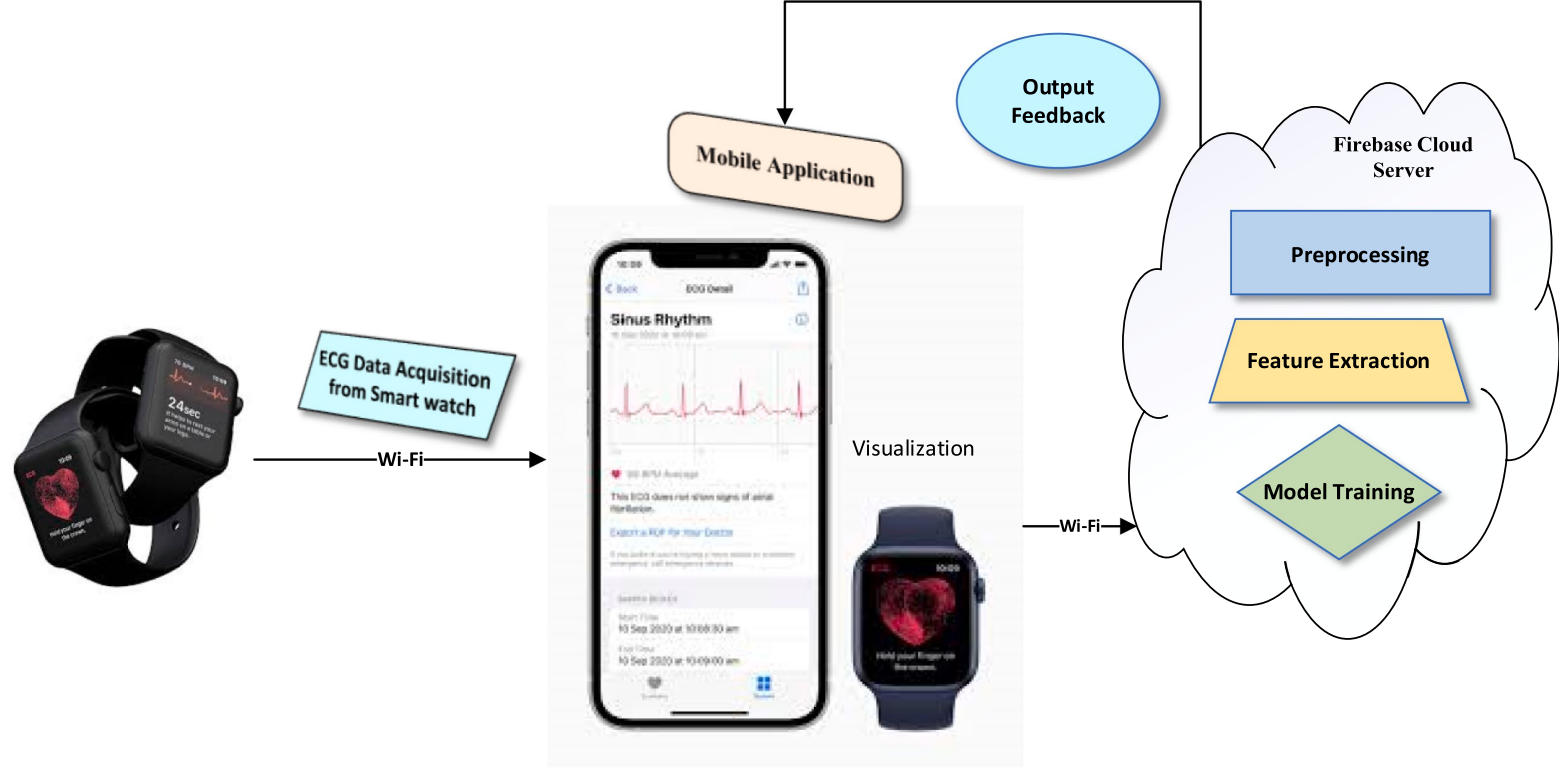
Hardware architecture of the Transformer-based ECG classification model.

Once trained, the optimized model is intended for future deployment on mobile devices, where it can support real-time ECG classification. The mobile application will be able to receive ECG signals and output classification results, identifying patterns such as Normal, atrial premature contraction (APC), premature ventricular contraction (PVC), Fusion beat, and other arrhythmic events. While the system is structured for scalability and real-time analysis, on-device inference and hardware-level performance optimization remain areas of future work to ensure clinical reliability and deployment in resource-constrained settings (Figure 1).

Workflow of the proposed ECG classification system, illustrating the integration of hardware components (wearable smart watch, mobile application, and cloud server) and data processing stages including signal acquisition, preprocessing, feature extraction, Transformer-based classification, and result delivery. The framework is designed to improve the accessibility of cardiac monitoring and supports the goal of enabling earlier detection of arrhythmias, though deployment and validation on real-world hardware remain subjects for future work.

### 3.1 Dataset description

**Figure 2:**
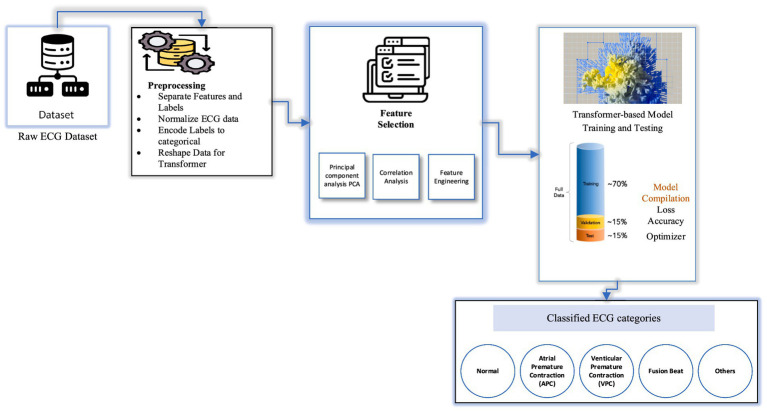
Architecture diagram of Transformer-based ECG classification model.

The dataset employed in this study comprises a collection of ECG recordings representing both normal rhythms and a range of arrhythmic conditions. All recordings are sampled at a consistent frequency, ensuring temporal uniformity across the dataset (20, 21). The dataset includes five clinically relevant classes: Normal, atrial premature contraction (APC), premature ventricular contraction (PVC), Fusion beat, and others, as illustrated in Figure 2. Although slightly imbalanced, it provides a diverse representation of common arrhythmic patterns. To ensure signal quality and reliability for downstream classification, preprocessing pipelines are applied to the raw ECG signals. This includes denoising, normalization, and segmentation steps, which help mitigate baseline drift, reduce motion artifacts, and standardize input lengths. These steps are essential to prepare the data for the attention-based Transformer model used in this study (6).

### 3.2 Data preprocessing

The preprocessing pipeline ensures the ECG signals are structured and standardized for input into the Transformer-based model. The key steps are as follows:

- Dataset loading and partitioning: The ECG dataset is first loaded and divided into training and testing subsets. Each row represents a single ECG sample, with the final column indicating the class label associated with the corresponding cardiac condition.
- Feature and label separation: The dataset is then split into feature matrices and target vectors. The features X_train, X_test → Contain Raw ECG features. While the Y_train, y_test → Contain corresponding class labels.
- Normalization: Given the variability in ECG signal amplitudes, normalization is applied to scale all feature values between 0 and 1. This mitigates amplitude-related noise, stabilizes the data distribution, and improves training convergence. The normalization is applied using the min-max scaling as shown in Equation 1:

(1) $$x{`}_{i}=\frac{x_{i}-\mu }{\sigma }\;$$

Where,

- xi is the normalized signal value.
- x_i_ is the original signal value.
- *μ* is the meaning of the signal segment.
- *σ* is the standard deviation of the signal segment.
- Normalization not only stabilizes input ranges but also accelerates model convergence and enhances classification performance by minimizing bias introduced by amplitude variations across different recordings.
- To analyze how well the normalized features represent different heartbeat categories, the t-distributed stochastic neighbor embedding (t-SNE) technique is applied. This dimensionality reduction method maps high-dimensional ECG features into a 2D space, allowing visual assessment of class separability prior to training. This step is particularly valuable for evaluating whether the features preserve inter-class distinctions.
- Since the task involves multi-class classification, categorical labels are transformed into numerical representations using a label encoding technique. This conversion is essential for training the deep learning model, allowing loss functions and optimization routines to operate effectively on class indices.
- Transformer models require input in a sequence-based format. Thus, the ECG data is reshaped into a 3D tensor with the structure.
- Samples (batch size).
- Time steps (ECG sequence length).
- Feature (single ECG value per step).

The reshaping is illustrated in Equation 2:


(2) $$X_{\text{down}}[i]=X[i.n]$$

Where,

- X_down_[i] is downsampled signal at index i.
- X[i] is an original signal.
- n is the down sampling factor.

This reshaping enables the model to process ECG signals as temporal sequences, ensuring that temporal dependencies and waveform dynamics are preserved during training. It aligns the data structure with the self-attention mechanism used by Transformers, which excels in modeling long-range dependencies without relying on fixed kernel sizes.

#### 3.2.1 ClassLabels

The dataset includes five distinct heart rhythm categories, each representing a specific type of arrhythmia or normal pattern:

- Normal: Represents a healthy, regular heart rhythm.
- Atrial premature contraction (APC): Premature beats originating from the atria, indicating irregular early electrical activity.
- Premature ventricular contraction (PVC): Extra systolic beats that originate in the ventricles, often associated with more serious cardiac conditions.
- Fusion beat: A waveform resulting from the combination of normal and abnormal heart contractions, leading to a hybrid signal.
- Other: Patterns that do not clearly fall into any of the above categories, encompassing miscellaneous or undefined anomalies.

Through the implementation of these preprocessing techniques, the ECG data is sanitized, segmented, and properly formatted before being fed into the Transformer-based classification model ensuring more accurate identification of a wide range of heart conditions.

### 3.3 Feature extraction techniques used in proposed model

Feature selection enhances model performance by identifying critical patterns and discarding irrelevant or less useful signal components (12, 13). After data preprocessing, multiple feature selection techniques are applied to ensure that only the most relevant features are retained for classification. The techniques used for feature extraction and selection include:

- Principal component analysis (PCA): A dimensionality reduction technique that transforms a set of potentially correlated variables into a smaller set of uncorrelated principal components, preserving the majority of the data’s variance.
- t-distributed stochastic neighbor embedding (t-SNE): A nonlinear dimensionality reduction technique primarily used for visualizing high-dimensional data in 2D or 3D.
- Correlation analysis: Used to detect and eliminate redundant features that show strong inter-feature correlation but do not contribute independently to classification performance.
- Feature engineering: The process of generating new, domain-relevant features derived from existing data to improve model accuracy.

While PCA and correlation-based feature selection significantly improved classification performance, their clinical interpretability remains limited. The principal components produced by PCA are linear combinations of original ECG features and, while they effectively capture statistical variance, they do not directly correspond to established clinical indicators such as P-wave duration, QRS complex width, or T-wave inversion. This raises uncertainty about whether the most influential features in the model’s predictions align with clinically accepted diagnostic markers used by cardiologists. This limitation underscores the need for future research that incorporates clinically annotated datasets and domain-informed feature selection strategies. Such efforts could bridge the gap between deep learning representations and clinically meaningful interpretations, improving trust and applicability in real-world diagnostic settings.

**Figure 3:**
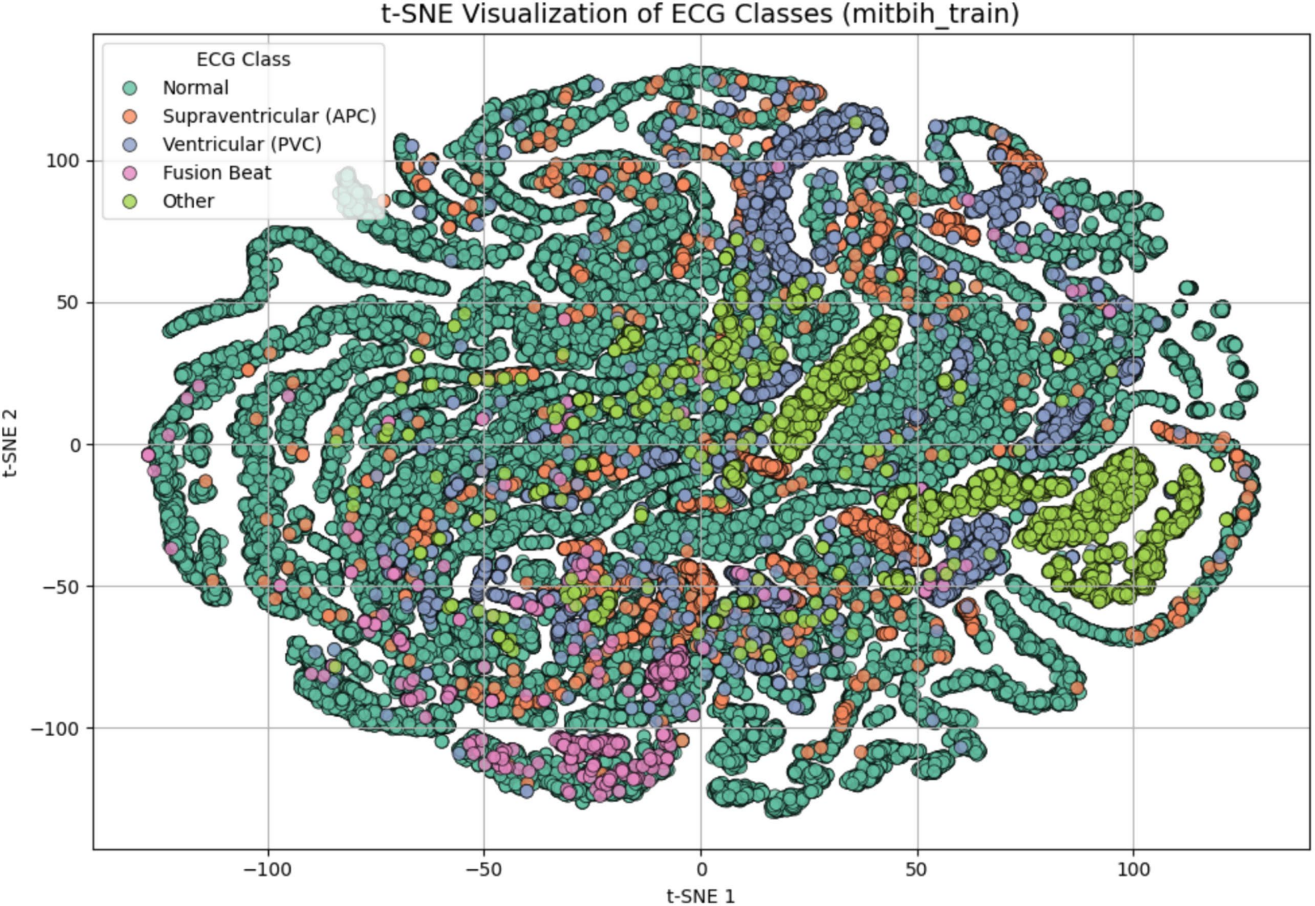
t-SNE visualization of ECG signal features after dimensionality reduction and preprocessing. Each point represents one ECG sample projected in a 2D space, colored by class label: Normal, supraventricular (APC), ventricular (PVC), fusion beat, and other. The visualization demonstrates that the extracted features possess natural class separation, indicating their suitability for classification.

To evaluate the discriminative quality of the extracted features before model training, we applied t-distributed Stochastic Neighbor Embedding (t-SNE) to the preprocessed dataset. As shown in Figure 3, the resulting 2D embedding reveals distinct clustering patterns for most arrhythmia types. This indicates that the features refined through PCA and correlation analysis retain sufficient discriminatory power for effective classification. The visual separation also validates the structure of the input space before learning begins, providing insight into class overlap and guiding model architecture decisions.

### 3.4 Transformer-based model training and testing

The Transformer-based model is trained on reshaped ECG input, where each sample represents a time-series sequence of cardiac electrical activity. The input data is formatted as a two-dimensional matrix, with dimensions corresponding to the sequence length and the feature dimension. The sequence length reflects the number of time steps (i.e., signal samples) in each ECG segment. The feature dimension represents the amplitude of the ECG signal at each time step, typically one-dimensional for raw ECG traces. This sequential structure is well-suited for Transformer architectures, which rely on self-attention mechanisms to capture long-range dependencies and temporal relationships in the input. Positional encodings are incorporated to retain temporal order information, as the Transformer lacks inherent recurrence or convolution. The model is trained using supervised learning, where ECG signals are paired with corresponding class labels (e.g., Normal, APC, VPC, Fusion Beat, Others). Training includes the use of optimized loss functions (e.g., sparse categorical cross-entropy), regularization techniques such as dropout, and hyperparameter tuning (e.g., number of attention heads, embedding dimensions, and learning rate) to improve generalization and prevent overfitting.

Tables 2, 3 illustrate the detailed architecture of the model, including the layer-wise parameters used in training. To assist with the initial level of feature extraction, the model incorporates an optional Dense Layer containing 64 neurons. This layer acts as a feature extractor, transforming the original input into a high-dimensional space (22). As a result, it highlights underlying steady-state patterns in ECG signals and enhances the model’s ability to recognize complex patterns in subsequent layers. Notably, no activation is applied in this Dense Layer, ensuring that the transformation remains linear (23, 24). After passing through the Dense Layer, the data undergoes a crucial reshaping step. This step resizes the input dimensions to be compatible length and an embedding dimension of 64, optimizing it for processing within the core Transformer block.

**Table 2:** Layer structure and parameters used in proposed model.

Layer type	Layer name	Parameters	Description
Input layer	Input	Input_shape = (X-train. Shape [1], 1)	Accepts input data reshaped to have one channel.
Flatten layer	Flatten	None	Flattens the input into a 1D array for initial processing.
Dense layer	Dense	Units = 64	Fully connected layer for initial feature extraction
Reshape layer	Reshape	Target-Shape = (−1, 64)	Reshapes the output to prepare it for the Transformer block.
Transformer block	TransformerBlock	Embed_dim = 64, num_heads = 4, ff_dim = 64	Custom layer implementing multi-head self-attention and feed-forward networks.
Global average pooling	GlobalAveragePooling1D	None	Reduce the output sequence to a single vector by averaging.
Output layer	Dense	Units = num_classes, activation = ‘softmax’	Final layer for classification, providing class probability.

**Table 3:** Transformer block breakdown of the proposed model.

Component	Parameters	Description
Multi-head attention	Num_heads = 4, key_dim = 64	Computers attention scores for different subspaces of the input.
Feed-forward network	Dense_layers: [64, 64]	It consists of two dense layers with a ReLU activation in between.
Layer normalization	Epsilon = 1e-6	Normalize the output for better training stability.
Dropout	Rate = 0.1	Regularization to prevent overfitting, applied after attention and feed-forward layers.

The core component of the model is the Transformer Block, which is specifically designed to capture temporal dependencies in ECG signals. This block begins with a Multi-Head Attention mechanism consisting of four heads and an embedding size of 64. These attention heads allow the model to process multiple time segments simultaneously, capturing both local and global features within ECG signals. This capability is crucial for identifying arrhythmia, as different time steps may contribute to abnormal heart rhythms.

To further refine feature extraction and visualization, the model leverages t-SNE after training. T-SNE is applied to the high-dimensional feature representations extracted by the Transformer blocks, providing an interpretable 2D visualization of how ECG patterns are separated based on different heart conditions. This technique helps assess how well the model distinguishes between normal and abnormal heartbeat, enhancing its explainability in real-world applications.

The self-attention mechanism for each head is computed in Equation 3:


(3) $$\text{Attention}\;(Q,K,V)=\text{softmax}\;(\frac{QK^{T}}{\surd \mathrm{dk}}\;)\;V$$

Where,

- Q = W^Q^ X, K = W^k^X and V = W^v^X are the query, key and value matrices.
- d_k_ is the dimension of Key vector.
- W_Q_, W_k_, and W_v_ are learnable weights matrices.
- QK^T^ is Dot product of the query and key matrices.
- Softmax ensures attention weights sum to 1.
- Scaling by √d_k_ helps with gradient stability.

For multi-head attention as shown in Equation 4:


(4) $$\text{MultiHead}\;(Q,K,V)=\text{Concat}\;(\mathrm{hea}d_{1},\ldots \ldots ,{\text{head}}_{h})\;\mathrm{Wo}$$

Where,

- h is the number of attention heads.
- head_i_ is output of the i-th attention head.
- W^o^ is an output weight matrix and h is the number of heads.

For feed-forward network (FFN):

Each transformer layer includes a position-wise FFN:


(5) FFN(x)=ReLU(xW1+b1)W2+b2

Where,

- W1, W2 are weight matrices for 2 linear layers.
- b_1_, b_2_ are bias terms for each layer.
- ReLU activation function applied after the first linear transformation.

For layer normalization and dropout:

After each attention and FFN block, layer normalization and dropout are applied:


(6) $$\text{LayerNorm}(x)=\frac{x-\mu }{\sqrt{\sigma^{2}+\epsilon }}.\gamma +\beta$$

Where,

- *μ* is the meaning of x.
-
σ ^2^ is the variance.
- ɛ is a small constant for numerical stability.
- *ϒ*, *β* are learnable parameters.

Following the attention mechanism, the output proceeds through a Feed-Forward Neural Network (FFN) which comprises of two Dense Layers. The first Layer again makes the function non-linear by using the ReLU activation function thus enabling the model to detect higher order compounding in the data. The second layer scales the output back to the embedding size of 64 needed for attention computations. This is further added by layer Normalization that settles the training process as well as Dropout that discards some neurons at random to avoid overfitting. To address the issues of high dimensionality of the data in the model with important features preserved, the model uses Global Average Pooling Layer. This layer pools the learned features over the time steps making it easy to work on an informed representation of the entire sequence.

The output from the transformer encoder is passed to a fully connected layer for classification, where softmax activation is used to assign probabilities to each ECG class as shown in Equation 7:


(7) Y=softmax(ZWc+bc)

Where,

- Z is the output from the encoder.
- Wc is the weight matrix.
- b_c_ bias terms for the classifier.

The output of the layer is feed to the Dense layer and a Softmax activation function is used. This last step computes probability for each of the five ECG classes which makes it possible for the model to perform multi-class classification. The model’s prediction is based on the maximum probability, which shows to which category the ECG signals belong, thus helping to diagnose arrhythmia correctly.

## 4 Results and evaluation metrics

The Transformer-based model’s performance was measured using various metrics to provide a comprehensive evaluation of its classification capabilities. The model achieved a final validation accuracy of 97% after 10 epochs, reflecting strong generalization on unseen data.

**Figure 4:**
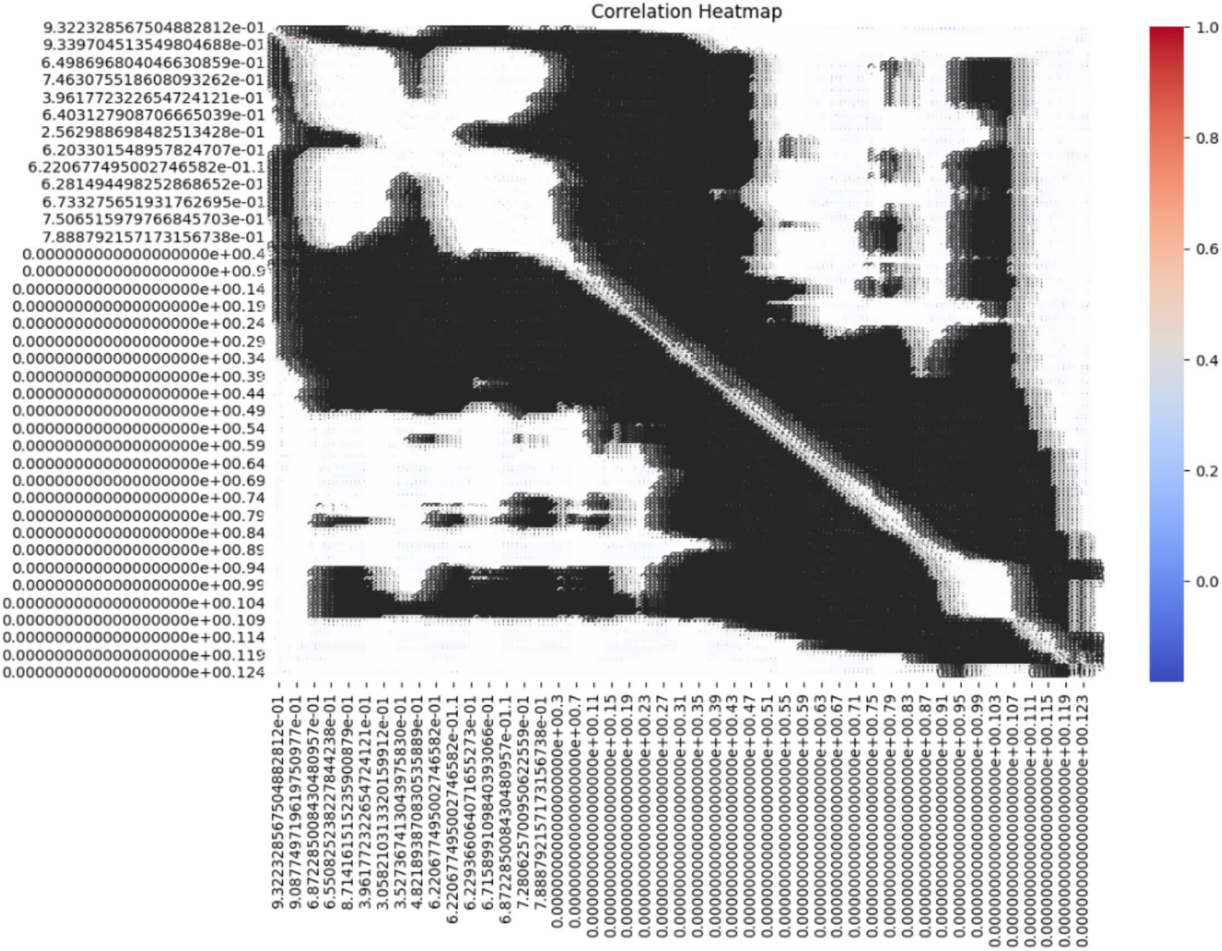
Correlation heatmap of the ECG dataset.

The correlation heatmap in Figure 4 depicts relationships among ECG features. Strong correlations (values close to 1 or −1) suggest redundancy, which guided the feature selection process using PCA. Features with low correlation were preserved to retain signal diversity. These insights helped reduce dimensionality while maintaining important clinical features. In this heatmap, every cell indicates the correlation between the two features of the bioinformatics dataset based on a coefficient varying between −1 to 1. Here, a value close to 1 reveals positive correlation, which makes one feature dependent on the other, whereas if one rises the other is also likely to rise. On the other hand, the value will be near −1, if the features are negative, thus suggesting that one of the features increases the other is likely to decrease (25). The heatmap uses a color gradient where darker colors signify higher positive correlation, lighter color signify low or negative correlation and black areas signify low correlation. Since each feature is compared to itself on the diagonal of the heatmap, it is obvious that the correlation between features would be 1. Some blocks in the heatmap contain areas with a clearly higher correlation, that can be attributed to groups of features that likely possess similar characteristics or possibly act in concert to manifest certain patterns in the ECG signals (26). Some blocks in the heatmap contain areas with a clearly higher correlation, that can be attributed to groups of features that likely possess similar characteristics or possibly act in concert to manifest certain patterns in the ECG signal. These observations indicated the possible redundancy or relevance of feature groups and might be helpful for the feature selection or dimensionality reduction (27). The lighter-colored areas or the areas with correlations near zero show that the features of these regions are least dependent on each other. Such features may be valuable for capturing some of the temporal qualities of the ECG signals that may be essential for the classification of arrhythmias. The heatmap analysis may show how various features related to arrhythmia are related to each other based on the pattern analysis. For instance, some attributes might appear to be more effective in identifying sorts of cardiac pathologies, knowledge of which can help to determine the model’s architecture.

**Figure 5:**
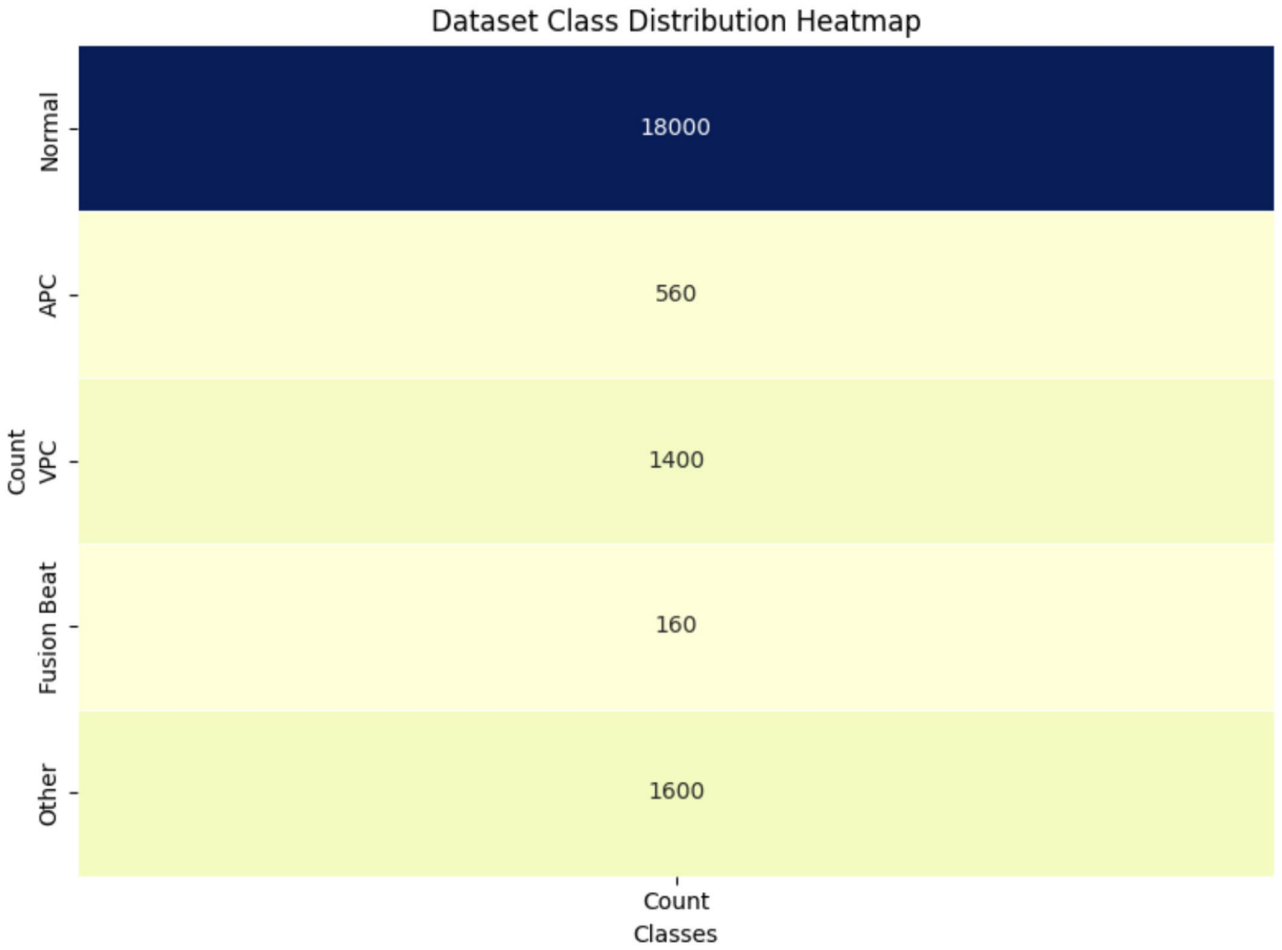
Class distribution heatmap of ECG dataset.

Figure 5 visualizes class imbalance in the dataset. The “Normal” class dominates with 18,000 samples, compared to 560 for APC and 1,400 for VPC. This imbalance motivated the use of augmentation and class-weighted training to prevent overfitting toward the majority class and improve minority class detection. The above figure provides the visual representation of the class distribution in the dataset, offering a clear view of the count of samples in each category (28). By using a heatmap, it emphasizes the significant class imbalance where the Normal class has a much larger sample size compared to other classes like APC, VPC, Fusion Beat and others. This disparity may impact the model’s performance, potentially leading to bias toward the majority class during training.

**Figure 6:**
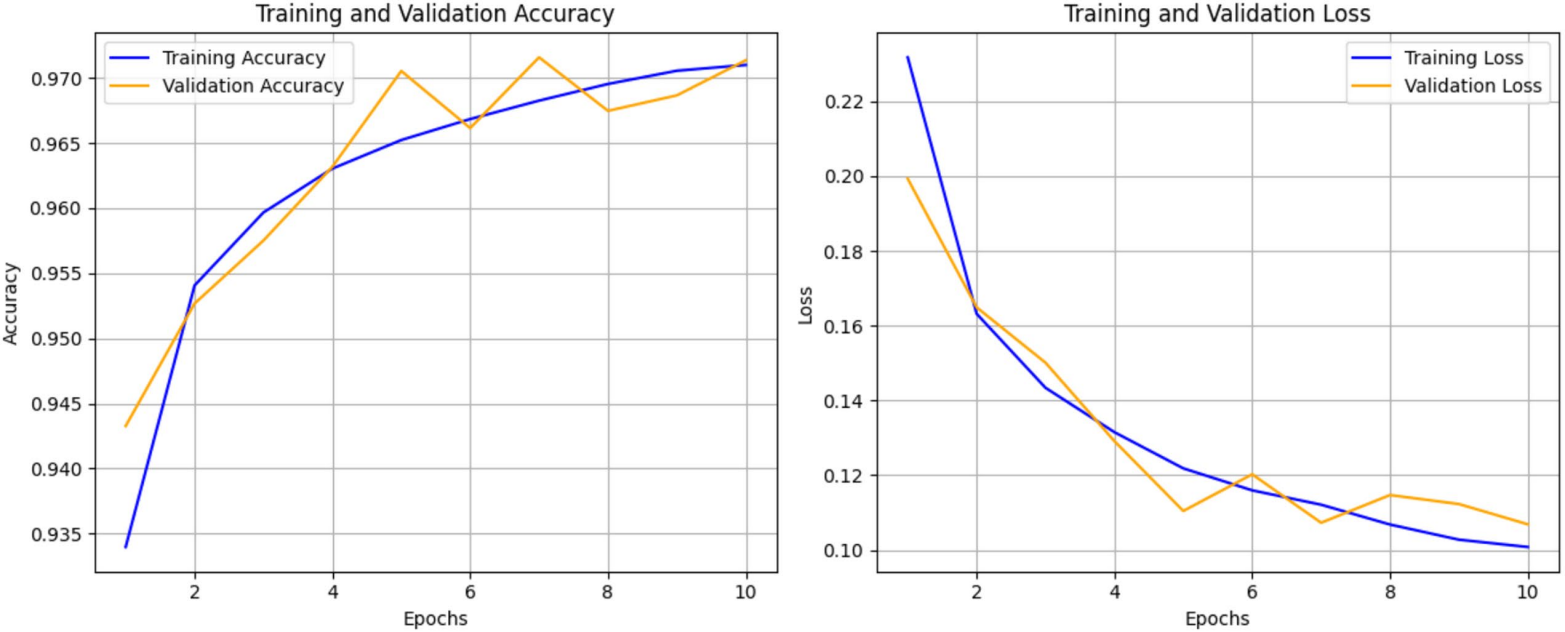
Training and validation accuracy and loss over 10 epochs of ECG classification model.

Figure 6 shows the progression of training and validation accuracy/loss over 10 epochs. Accuracy steadily increased while loss decreased, with both curves converging by the 10th epoch. This indicates minimal overfitting and efficient learning. This trend suggests that the model is learning effectively and improving its predictions over time (29). The closeness of the training and validation accuracy curves indicates minimal overfitting, as the validation accuracy closely follows the training accuracy. The right curve, loss curve, augments downward with the training time, showing less error of prediction. The training and validation losses converge closely by the final epoch, indicating stable performance, which is additional evidence of model performance on unseen data. But the early epoch oscillates a bit, and this could mean the model is making changes to the learning rate or complexities in some classes. All these plots show that the model performed very well and with little overfitting which implies that there was good or sufficient balancing between the training and the validation accuracy models. This model appears well-optimized for this dataset, though further comparisons with baseline models are required to confirm its superiority, since both the accuracy and the loss rate converge quite steadily.

### 4.1 Quantifying the impact of PCA

While both principal component analysis (PCA) and t-distributed stochastic neighbor embedding (t-SNE) were used in the study, their individual contributions were distinctly different. PCA was applied as a dimensionality reduction technique prior to training, aiming to eliminate redundancy and retain the most informative features. To evaluate its effectiveness, an ablation experiment was conducted where the Transformer model was trained once with PCA and once without PCA, using the same training configuration (Table 4).

**Table 4:** Impact of PCA on the model performance.

Model setup	Accuracy	AUC
Without PCA	92.3	0.91
With PCA	97.1	0.96

These results confirm that PCA significantly improved model performance by reducing feature noise and enhancing separability in the feature space. In contrast, t-SNE was used exclusively for visualization to illustrate class-wise separability and decision boundaries in a reduced feature space. It was not used during training and did not influence model accuracy directly.

**Figure 7:**
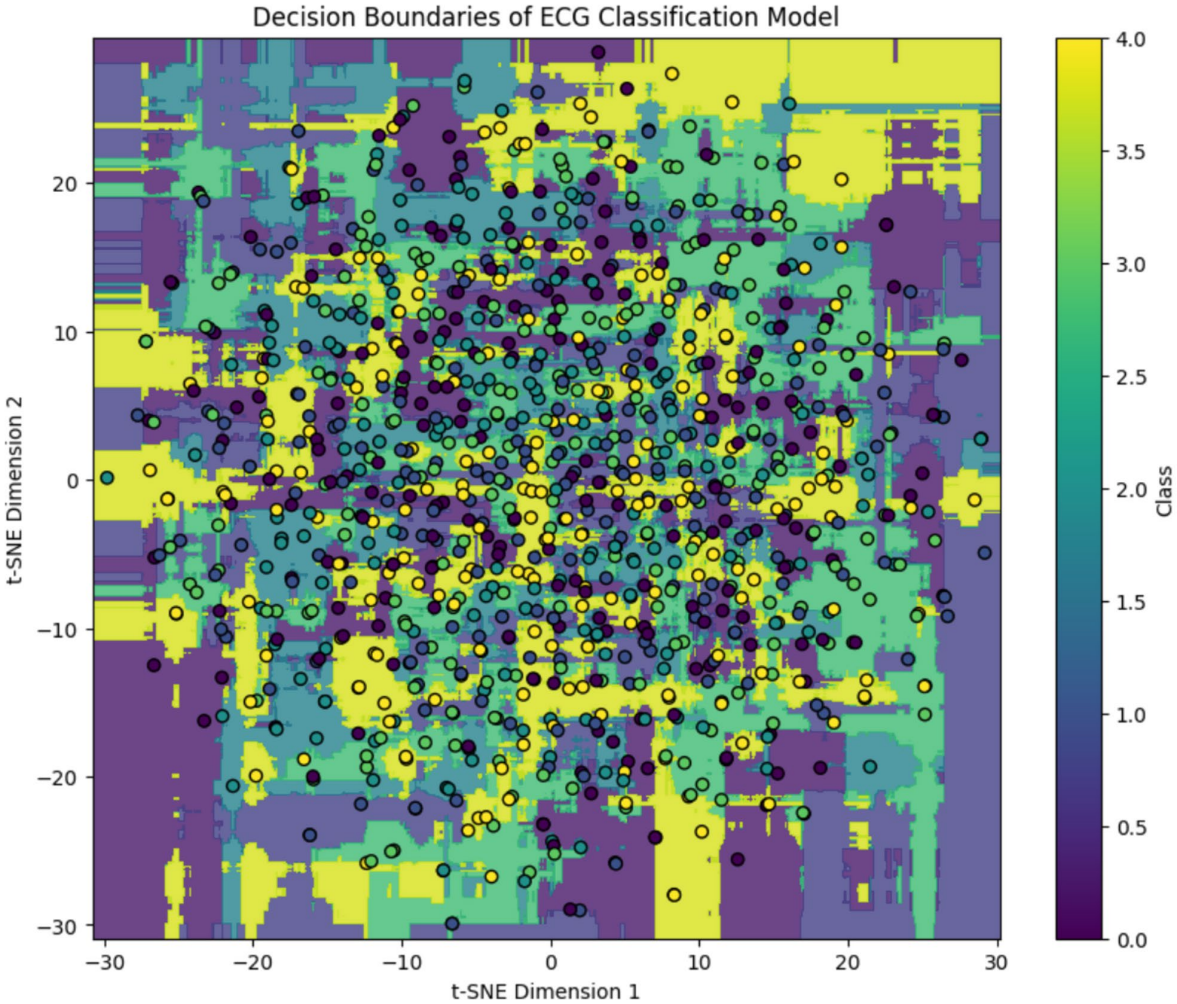
Post-training t-SNE decision boundary visualization of the ECG classification model. Background regions indicate model-predicted class clusters, and colored circles represent projected ECG samples. While distinct clusters emerge for dominant classes, class overlap remains in minority arrhythmias.

To interpret the model’s behavior after training, we applied t-distributed Stochastic Neighbor Embedding (t-SNE) to the learned feature embeddings and visualized the decision boundaries for each ECG class. As shown in Figure 7, the background color represents the class regions predicted by the trained Transformer-based model, while the overlaid dots indicate actual test samples projected into the 2D t-SNE space. The clear separation in some regions particularly for classes like “Normal” and “Fusion Beat” indicates strong class-specific learning. However, overlapping regions involving “APC” and “VPC” reflect residual class confusion, consistent with class imbalance and similar signal morphology. This visualization confirms that the model has successfully learned a meaningful embedding space for ECG classification, while also highlighting opportunities for further refinement.

**Figure 8:**
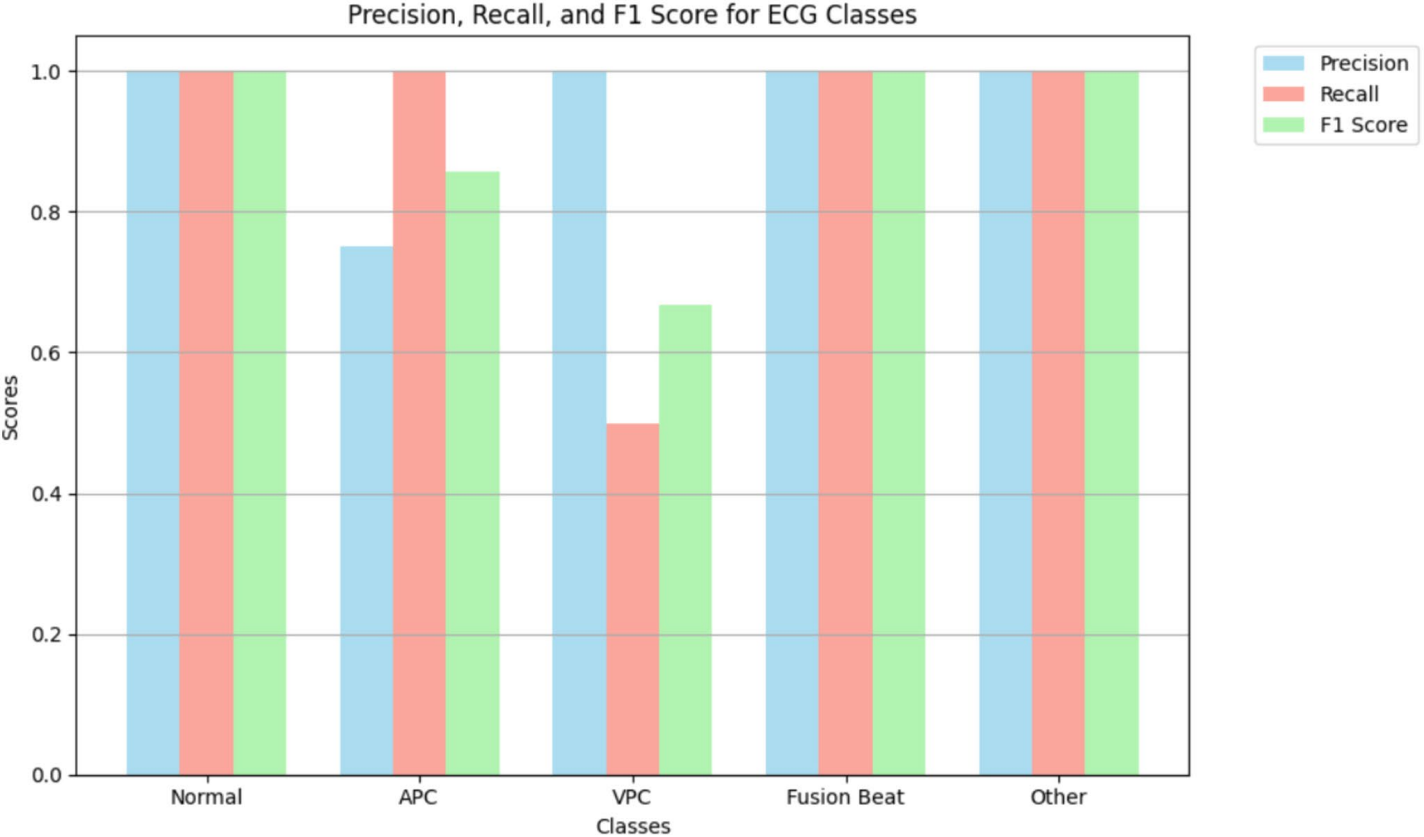
Highlighting model performance across various arrhythmia types.

Figure 8 illustrates the precision, recall, and F1 score for each ECG class, reflecting the model’s classification performance across different arrhythmia types. The results show that the model achieves near-perfect precision and F1 scores for the “Normal,” “Fusion Beat,” and “Other” categories, indicating excellent classification for these classes. For the *Atrial Premature Contraction* (APC) class, the model demonstrates strong recall (100%), suggesting it detects nearly all APC instances. However, the precision is relatively low, resulting in an F1 score above 85%. This implies the model over-predicts APC, likely due to its confusion with similar classes such as Normal. The *Ventricular Premature Contraction* (VPC) class exhibits the weakest performance, with noticeably lower recall and F1 score. This may be due to class imbalance and the morphological similarity of VPC to APC and Fusion Beat in ECG waveforms particularly within the QRS complex, where overlapping features can confuse the classifier.

Interestingly, the VPC class shows a perfect AUC (1.00), indicating that the model is capable of ranking VPC instances correctly. However, the low recall suggests that classification thresholds or insufficient representation in the training data may limit actual detection. This highlights the need for possible threshold adjustment or targeted data augmentation.

**Figure 9:**
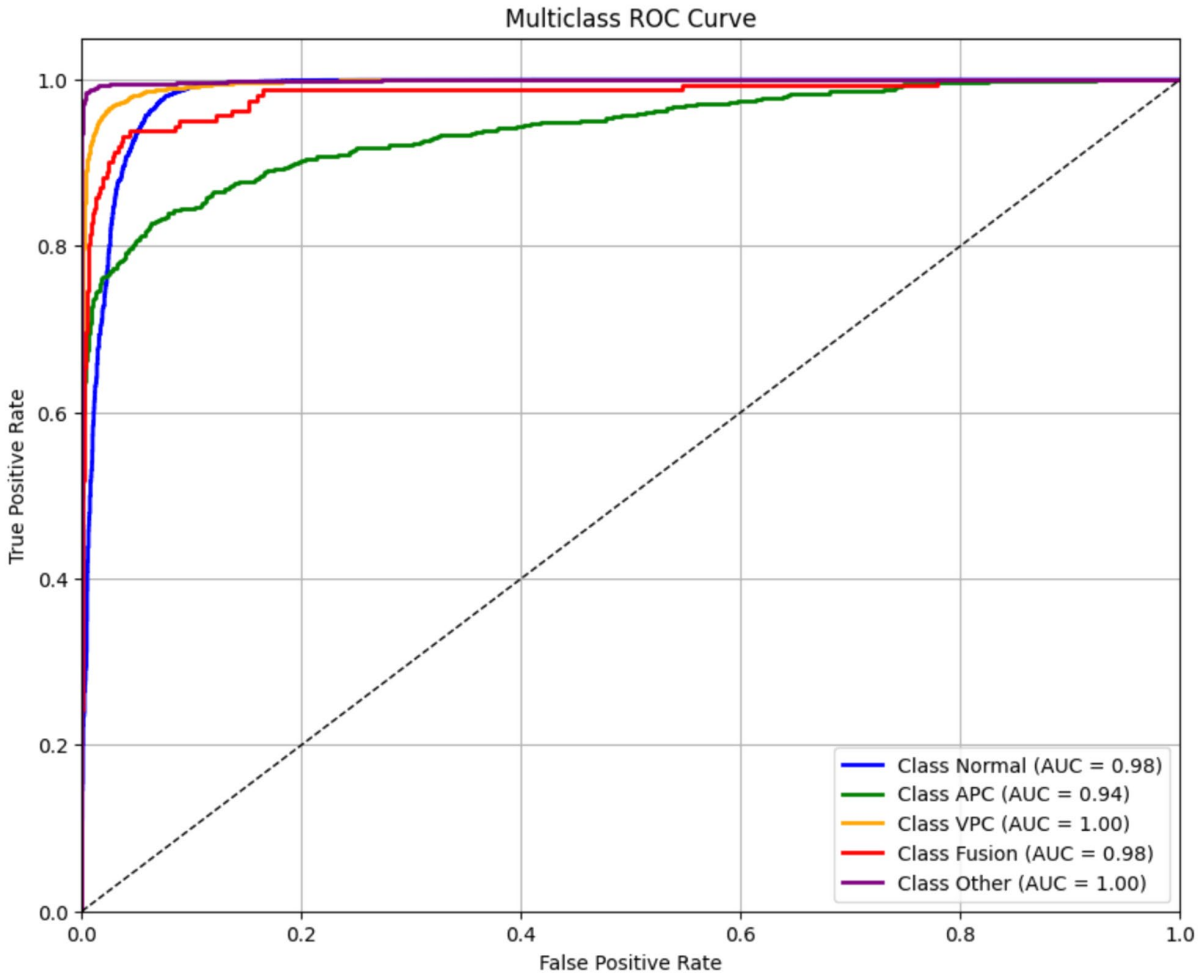
Receiver operating characteristic (ROC) curves for ECG classes.

Figure 9 displays the ROC curves for each ECG class in the classification model, showing the trade-off between the true positive rate (TPR) and false positive rate (FPR) at various classification thresholds. The ROC curve is a standard diagnostic tool to evaluate the model’s ability to distinguish between different classes. The area under the ROC curve (AUC) provides a scalar measure of this discriminative ability. AUC values closer to 1.0 indicate excellent class separability, while values near 0.5 suggest random guessing. In this model, all ECG classes achieved high AUC scores, reflecting strong performance:

- Normal: 0.98.
- APC: 0.94.
- VPC: 1.00.
- Fusion Beat: 0.98.
- Other: 1.00.

These results indicate that the model is highly capable of distinguishing between the different rhythm types, even for more challenging arrhythmias like APC and VPC. Despite some misclassifications seen in the confusion matrix and F1 scores (particularly for VPC), the high AUC values suggest that the model’s ranking ability is robust. This discrepancy implies that classification thresholds, class imbalance, or feature overlap might be affecting precision and recall, rather than the model’s core ability to separate classes. Therefore, further improvements could be made through threshold tuning, class-specific loss weighting, or augmentation strategies, rather than architecture changes:


(8) TPR(t)=TP/TP+FN,FPR(t)=FP/FP+TN

The final AUC score is computed by integrating the area under the ROC curve.

**Figure 10:**
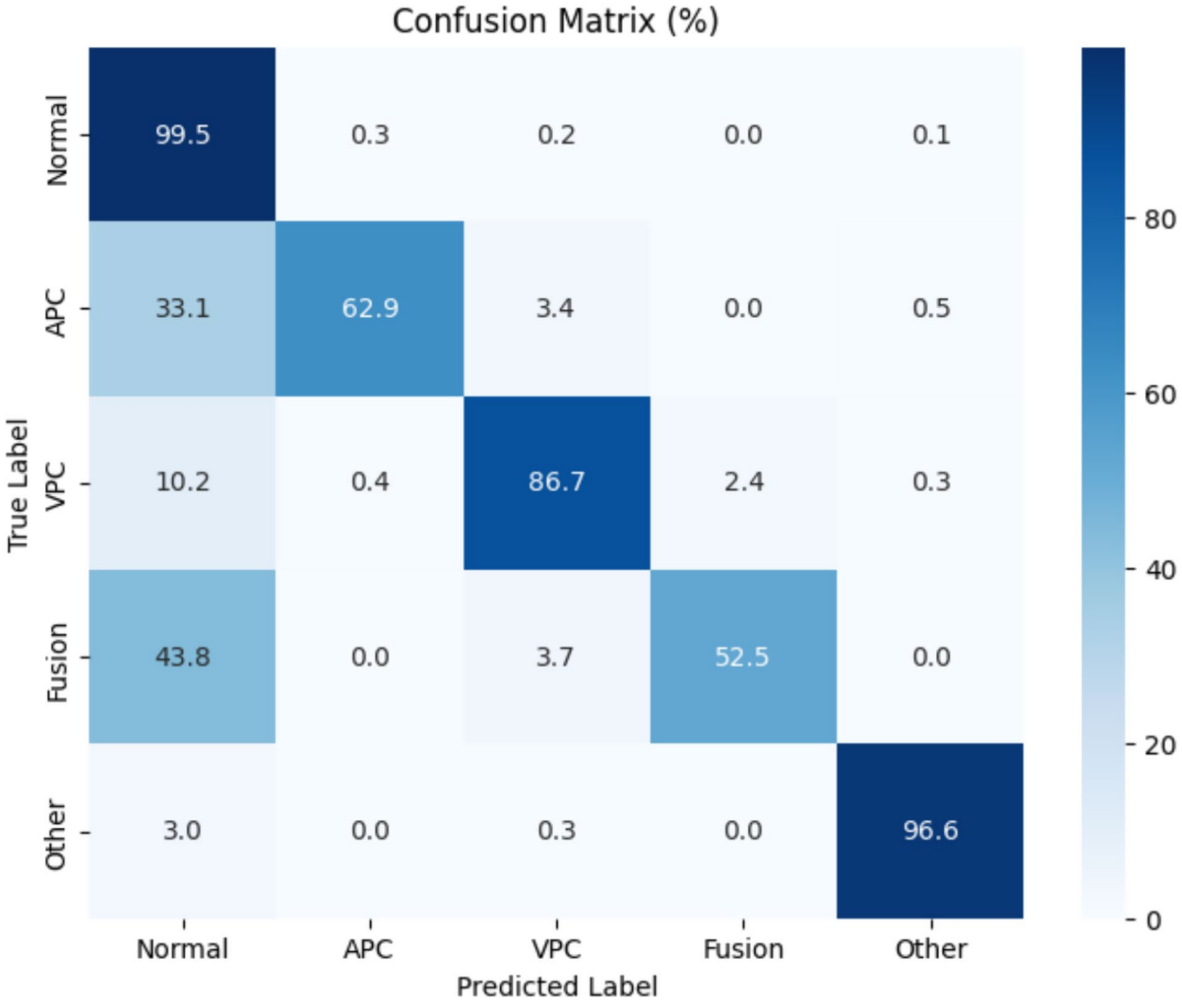
Confusion matrix for Transformer-based ECG classification model across classes.

Figure 10 presents the normalized confusion matrix, providing a detailed view of the model’s classification performance across ECG rhythm categories: Normal (0), APC (1), VPC (2), Fusion Beat (3), and Other (4). Each cell indicates the percentage of instances from a true class (rows) predicted as a certain class (columns). Diagonal values represent correct classifications, while off-diagonal values indicate misclassifications.

The matrix shows excellent performance on the Normal class, with 99.5% of samples correctly classified, reflecting the model’s high sensitivity and specificity for detecting normal heartbeats. The “Other” category also shows strong results, with over 96% correctly identified.

However, some confusion is evident among arrhythmic classes:

- APC is often misclassified as normal (33.1%), despite a high recall.
- Fusion Beat is frequently predicted as normal (43.8%), suggesting difficulty in distinguishing Fusion morphology from typical ECG rhythms.
- VPC shows good accuracy (86.7%), but a small portion is misclassified as normal (10.2%) or fusion (2.4%).

These misclassifications likely arise from morphological similarities in the QRS complexes and overlapping waveform features across arrhythmia types. In particular, the confusion between APC and Normal, and Fusion and Normal, may stem from subtle variations in signal patterns that challenge the model’s feature extractor.

To enhance class separability, especially for VPC and Fusion, future work could focus on improving the feature extraction pipeline, incorporating class-specific augmentation, or using contrastive learning techniques to better differentiate similar waveform classes in the learned embedding space.

The model was trained to minimize sparse categorical cross-entropy loss, which quantifies the difference between the predicted probability distribution ppp and the true distribution qqq. The loss function is defined in Equation 9:


(9) Loss=−ΣNi=1qilog(pi)

Where,

- *N* is the number of classes.
- q_i_ is 1 for the correct class and 0 otherwise.
- p_i_ is predicted for class I.

Overall accuracy, which is the ratio of correctly predicted instances to the total number of instances, is defined as:


(10) Accuracy=True Positive+True Negatives/Total Instances

These metrics help to evaluate the model’s performance in each class:

Precision measures the accuracy of positive predictions:


(11) $$\text{Precision}=\frac{\;\text{True Positives}}{\text{True Positives}+\text{False Negatives}}$$

Recall measures the model’s ability to capture all relevant instances:


Recall=True Positives/True Positives+False Negatives

F1-score is the harmonic mean of precision and recall, balancing the two metrics:


(12) F1−Score=2∗Precision∗Recall/Precision+Recall

When evaluating the model with respect to precision, recall and F1-score as well as the analysis of the confusion matrix, the model would be strong in predicting classes that make the majority such as 29 K, 44 K thus indicating the areas that would require improvement in the minority classes such as APC and VPC. The model architecture could also be improved further and overspecification of hyperparameters could be done to achieve a better balance among all classes.

## 5 Comparative evaluation of transformer variants

To demonstrate the effectiveness of our proposed model, we compared its performance with other state-of-the-art Transformer-based ECG classifiers, including ECG-BERT, time series transformer (TST), and Informer. These models were selected based on their recent use in biomedical signal processing and sequential data tasks.

Table 5 summarizes the comparative performance of various state-of-the-art Transformer-based models applied in biomedical signal classification. Among them, ECG-BERT, Informer, and time series transformer (TST) demonstrate strong performance on arrhythmia detection tasks, with AUC scores ranging from 0.94 to 0.95. These models leverage attention mechanisms to effectively model temporal dependencies within ECG signals. MN-STDT model proposes a brand-new multimodal framework, where chest X-ray spatial features and EHRs temporal features are combined, with an AUC of 0.8620 in in-hospital mortality prediction of heart failure. Despite not being directly applicable to ECG classification, MN-STDT demonstrates the increased nexus of multimodal Transformer models in clinical research and their ability to perform more context-aware predictions. In their turn, the suggested Transformer model of the present research, based on the use of the PCA-based feature selection, engineered representations as well as t-SNE visualization, attains higher performance, with an accuracy ratio of 97.1, F1-score rate of 0.95 and the value of AUC equals to 0.96. It suggests that, besides the overall success of the Transformer backbone at modeling ECG sequences, the well-optimized preprocessing, dimensionality reduction, and hyperparameters tuning play a central role. As opposed to other models, the proposed one has a high degree of interpretability and generalization to different classes of ECG, indicating its strong potential to be broadly integrated into the clinical routine in automated pipelines of ECG analysis.

**Table 5:** Comparison with Transformer-based and SOTA ECG models.

Model	Architecture	Accuracy	F1-score	AUC	Reference
ECG-BERT	Pre-trained transformer (BERT-based)	94.6	0.92	0.94	(36)
Time series transformer (TST)	Encoder-only transformer with positional encoding	95.3	0.93	0.95	(37)
DRL-ECG-HF	DRL + Multi-instance learning + PER + SHAP	–	0.58	9.90	(38)
MN-STDT	Spatially and temporally decoupled transformer with multimodal fusion (CXRs + EHR)	–	–	0.86	(37)
Proposed transformer model	Transformer + PCA + Feature engineering	97.1	0.95	0.96	Current study

## 6 Ablation study of hyperparameter settings

**Figure 11:**
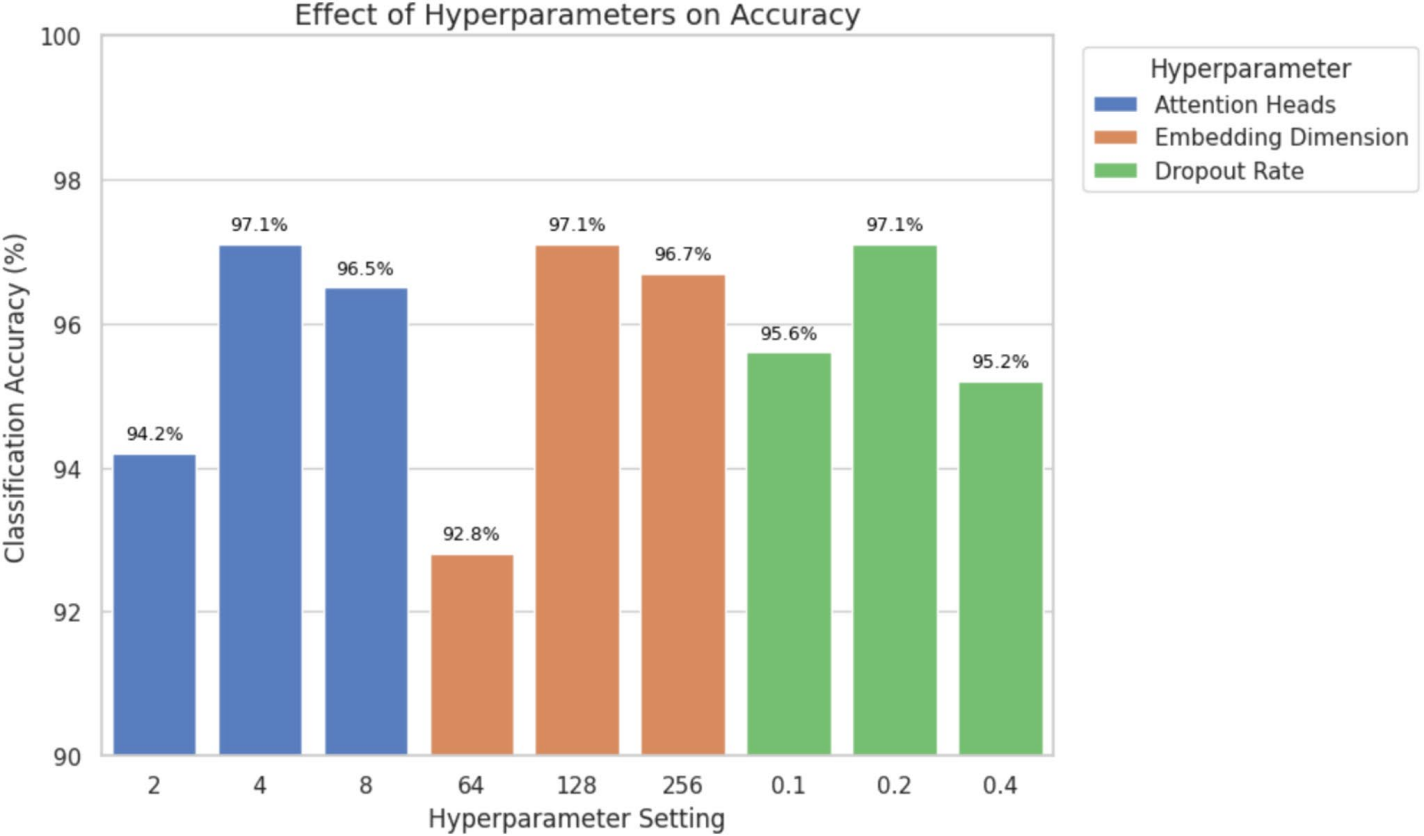
Ablation study showing the effect of attention heads, embedding dimension, and dropout rate on classification accuracy. Optimal performance was achieved with 4 attention heads, 128-dimensional embeddings, and a dropout rate of 0.2.

An ablation study was undertaken to assess the effectiveness of parameter ablation by varying the number of attention heads, the size of embedding dimension and dropout rate independently. This analysis aimed at finding the most optimal values that would offer classification accuracy and model complexity. Table 5 presents the classification accuracy and AUC values obtained by modifying one hyperparameter at a time while keeping the others constant (Figure 11).

The results demonstrate that using four attention heads and an embedding dimension of 128 achieved the highest classification accuracy and AUC without significantly increasing the computational cost. A dropout rate of 0.2 provided effective regularization, reducing the risk of overfitting while preserving performance. Higher dropout values (e.g., 0.4) led to underfitting, while lower values (e.g., 0.1) increased variance during training. These findings support the final hyperparameter configuration used in the proposed model and confirm that the selected values contribute meaningfully to improved classification outcomes, particularly for clinically relevant ECG classes.

## 7 Discussion

The transformer model as applied to the ECG has high classification accuracy across various classes of arrhythmias which implies that the model can handle temporal variability and complex morphologies of the ECG signals. By using self-attention, the model learns dependencies that are long-range without constraints to fixed-size temporal windows and recurrent architecture. This is because it can accommodate ECG sequences with different sequences and dynamics; this is a common feature in clinical data. Consequently, the sensitivity to the slight variation of the waveforms which is important in identifying the classification of arrhythmia is better enhanced on the model. Although CNN-based models have shown strong results in ECG analysis and remain widely used in clinical and research settings, their reliance on local receptive fields limits their capacity to capture long-range dependencies. Transformers overcome this by using self-attention mechanisms that dynamically model relationships across the entire signal length. Conversely, Transformer global attention mechanism better captures temporal dependencies to yield better classification results. Although model performance is one of the priorities, explaining the model still is a big challenge. Transformer-based models can be regarded as black boxes and even the presented techniques such as visualization of attention weights may provide some insight into the models, but this paper does not envisage an analysis of interpretability. Future research is advised to include implementations of explainability algorithms like attention mapping or SHAP analysis, seeking to make the inclusion of such systems more clinically acceptable and easy to adapt to.

Additionally, although architecture holds potential for integration into edge devices and wearable technologies, this study does not evaluate inference latency, computational resource requirements, or hardware deployment feasibility. As such, claims regarding real-time or mobile deployment are beyond the scope of this work. Future research may explore model simplification, quantization, or pruning strategies to enable deployment in resource-constrained environments, such as wearable health monitoring systems.

Overall, this study underscores the applicability of Transformer architectures to biomedical signal classification tasks, particularly ECG interpretation, and provides a foundation for future research focused on explainability, deployment, and clinical validation.

## 8 Limitations

Although the current Transformer-based ECG classification model shows promising results, several limitations must be acknowledged.

First, the dataset used for training and evaluation lacked significant diversity and exhibited class imbalance. While the model performed well on majority classes such as “Normal” and “Fusion Beats,” it underperformed on minority classes like “Ventricular Premature Contractions (VPC),” which had relatively few samples. This imbalance likely affected the model’s ability to accurately classify rare arrhythmias and limits its generalizability to diverse or unseen clinical scenarios.

Second, the transformer architecture is computationally intensive, both during training and inference. Memory and processing demand pose challenges for deployment in resource-constrained environments, such as mobile or wearable healthcare devices. This limitation impacts the model’s scalability and increases the cost and complexity of real-world implementation.

Third, interpretability remains a significant concern. Despite the theoretical advantages of attention mechanisms in revealing important features, Transformer-based models continue to function largely as black boxes. Current attention visualization techniques provide limited insight into the model’s reasoning, which hinders clinical trust and diagnostic transparency. Clinicians require explainable models to validate predictions and make informed decisions, and the lack of interpretability restricts practical adoption in healthcare settings. Finally, direct comparison with prior studies is constrained by inconsistencies in datasets, preprocessing pipelines, and evaluation metrics. Although Table 5 summarizes performance metrics and limitations of previous approaches, such comparisons should be interpreted cautiously due to differing experimental setups.

In summary, these limitations underscore key areas for future improvement, including addressing class imbalance, optimizing model efficiency for deployment, and enhancing model transparency. Addressing these challenges is essential to advance the clinical applicability of deep learning-based ECG analysis systems (Table 6).

**Table 6:** Limitations of various approaches used in ECG classification.

References	Model	Accuracies	Limitations of previous work	Limitations of current transformer model
Smith et al. (39)	Transformer-based model for ECG diagnosis	Evaluated by sensitivity, PPV, and detection of major abnormalities	Lower accuracy in detecting major abnormalities; higher false positives/negatives leading to reduced diagnostic reliability	Sensitive to ECG noise; misclassification of subtle abnormalities; requires large, annotated datasets; trade-off between sensitivity and specificity
Zhao et al. (40)	CNN-RNN (Deep Convolutional Neural Network – Recurrent Neural Network)	97.6% (for 2-s ECG segments)	Lacked real-time inference; limited performance in heart failure staging; complex feature extraction pipeline	Requires intensive preprocessing (segmentation, augmentation); limited capacity to capture long-range dependencies
Chithra et al. (41)	ANN-based Decision Tree	93.4%	Poor integration of clinical and ECG features; low model interpretability	High feature engineering cost; poor scalability to multilead/multiclass ECGs
Arabi et al. (19)	MSW-Transformer	Macro-F1 up to 77.85%	CNN only captures local patterns	Complex architecture, data-hungry sliding windows
Uğraş et al. (42)	CardioPatternFormer	Interpretable, multi-pathology	Opaque black-box models	May overfit attention map, needs clinical validation
Luo et al. (43)	Hierarchical Transformer	-	Single-scale Transformers	Multi-stage model is resource-intensive
Alghieth (44)	DCETEN	99.84% acc. (MIT-BIH)	Heavyweight transformer models	Still GPU-reliant despite pruning
Current study	Transformer Model	97%	N/A	High Computational demand, requiring advanced GPUs or TPUs, limited interpretability, challenging clinical transparency.

Lastly, generalizability remains a fundamental concern due to the homogeneity of the dataset, which was collected from a specific demographic using a single device type. ECG signals can vary across different populations, age groups, and acquisition devices, potentially affecting the model’s performance in diverse clinical settings. As a result, the effectiveness of the proposed model may be limited when applied outside the specific context in which it was trained.

To enhance generalizability and clinical robustness, future studies should aim to validate the model on datasets collected from multiple sources, encompassing both homogeneous and heterogeneous subject groups. This includes variations in age, ethnicity, health conditions, and recording hardware. Such external validation would provide a stronger basis for assessing the model’s adaptability and reliability in real-world clinical environments.

## 9 Future work

In subsequent studies, efforts will focus on enhancing the robustness, clinical reliability, and deployment readiness of Transformer-based models for ECG classification.

One key direction is addressing class imbalance, particularly for underrepresented arrhythmia types such as Ventricular Premature Contractions (VPC), which currently contribute to lower classification accuracy. Sensitivity to rare classes may be improved by applying techniques such as class-specific data augmentation, oversampling, and class-weighted loss functions.

Another priority is improving the diversity and representativeness of the training data. Incorporating ECG signals from a broader population encompassing different demographics, acquisition devices, and arrhythmia types can increase the model’s generalizability and reduce bias toward specific data sources or conditions.

To further improve diagnostic accuracy, future work may explore multimodal learning by integrating additional physiological signals such as heart rate variability, blood oxygen saturation, and blood pressure. These complementary modalities could enhance the feature space and provide more context for ECG interpretation.

Optimizing the model for deployment in resource-constrained environments, such as mobile or wearable devices, is also a critical focus. While Transformers offer high accuracy, their computational demands limit feasibility on low-power platforms. Future research will investigate lightweight Transformer variants, as well as model compression techniques such as pruning and quantization, to reduce inference costs while preserving clinical performance.

Finally, improving model interpretability remains a central challenge. Future studies will incorporate explainability techniques such as attention weight visualization, relevance mapping, and lead-wise contribution analysis. These tools can help clinicians better understand the basis for automated predictions, thereby increasing trust and promoting adoption in real-world healthcare settings.

## 10 Conclusion

The proposed Transformer-based ECG classification model demonstrates strong potential in accurately diagnosing multiple cardiac arrhythmias from raw ECG signals. Leveraging the self-attention mechanism inherent in Transformer architecture, the model effectively captures the temporal dependencies of ECG sequences and achieves high classification accuracy across several classes, including Normal, APC, VPC, Fusion Beats, and Others. These results confirm the suitability of attention-based models for analyzing the complex and sequential nature of biomedical time-series data.

A key contribution of this work is the demonstration that transformer models can serve as effective tools for ECG signal classification, providing clinically relevant outputs with high precision, recall, and F1-scores particularly for classes with ample training data. This suggests that such models can complement existing machine learning techniques in automated ECG interpretation.

In addition to performance, the model offers potential for integration into future clinical workflows, where automated ECG analysis can support healthcare professionals by reducing manual diagnostic load and improving consistency. However, several challenges remain before deployment in real-world settings. These include improving classification for underrepresented arrhythmia classes, validating the model across more diverse populations and device types, and enhancing model interpretability and computational efficiency.

Future work should focus on optimizing the model for broader generalization, incorporating multimodal physiological data, and adapting the architecture for deployment in resource-constrained environments such as wearable healthcare devices. With further development and clinical validation, Transformer-based models may play an important role in advancing automated, scalable, and accessible cardiac diagnostics.

## Data Availability

The original contributions presented in the study are included in the article/supplementary material, further inquiries can be directed to the corresponding author.
